# Anti-inflammatory mechanism of total flavonoids from *Polygala fallax* Hemsl. based on network pharmacology, molecular docking, and experimental validation

**DOI:** 10.3389/fimmu.2025.1690388

**Published:** 2025-10-07

**Authors:** Chunhong Liao, Hua Su, Fengzhen Li, Chenglong Wang, Sufang Yang, Zujie Qin, Ning Li

**Affiliations:** ^1^ International Zhuang Medical Hospital Affiliated to Guangxi University of Chinese Medicine, Nanning, China; ^2^ Guangxi Institute of Chinese Medicine and Pharmaceutical Science, Nanning, China; ^3^ Guangxi Key Laboratory of Traditional Chinese Medicine Quality Standards, Nanning, China

**Keywords:** inflammation, total flavonoids of *Polygala fallax* Hemsl., network pharmacology, molecular docking, potential target, RAW264.7 cells

## Abstract

**Objective:**

To explored the anti-inflammatory mechanisms of total flavonoids of *Polygala fallax* Hemsl. (PFHF) using network pharmacology, molecular docking, and cellular experiments.

**Methods:**

Key components, targets, and pathways of PFHF were identified via literature and network pharmacology, with molecular docking and dynamics simulations validating binding to therapeutic targets. RAW264.7 cells were treated with lipopolysaccharide (LPS) to establish inflammation, and groups included blank controls, LPS-induced models, prednisolone acetate, and low/high-dose PFHF. Cytokine levels (IL-6, TNF-α, IL-1β) were measured by ELISA, while immunofluorescence assessed protein expression post-PFHF treatment.

**Results:**

Six major active components were identified, alongside 44 active components, 1,178 inflammatory genes, and 18 target genes. Core targets included IL-6, TNF, IL1B, INS, and CASP3. Gene Ontology (GO) analysis linked these targets to protein localization, membrane rafts, and receptor activity. Kyoto Encyclopedia of Genes and Genomes (KEGG) pathways highlighted IL-17, TNF, and NOD-like receptor signaling. Molecular docking confirmed rutin’s strong binding to IL-6, TNF, IL-1β, INS, and CASP3. HPLC quantified rutin at 0.09 mg/mL. PFHF inhibited RAW264.7 proliferation with IC50 values of 206.32 µg/mL (24h) and 102.39 µg/mL (48h). High-dose PFHF reduced IL-6, TNF-α, and IL-1β (P<0.05) versus the model group. Immunofluorescence revealed elevated INS (P<0.05) and reduced CASP3 (P<0.01), iNOS, and Cox-2 (P<0.0001) in treated cells.

**Conclusion:**

PFHF exerts anti-inflammatory effects via IL-17 and TNF pathways, targeting IL-6, TNF-α, INS, IL-1β, and CASP3, mediated by rutin and other components.

## 1 Materials and instruments

### 1.1 Main materials

PFH tablet (purchased from Pharmacy of Guangxi International Zhuang Medical Hospital, batch No.: 20230901); Mouse macrophage RAW264.7 (purchased from Zhongsheng Beikong Biotechnology Co., LTD., identified by STR); INS antibody (Proteintech, 001048883), CASP3 antibody (Proteintech, 00136098), i-NOS antibody (Abcam, GR3240243-2), Cox-2 antibody (Abcam, 00136098) GR3381592-5), donkey anti-rabbit fluorescent secondary antibody (Abcam, GR3360238-2); CCK-8 kit (meilunbio, MA0218-3-Mar-05G); IL-6 mouse ELISA test kit (shfksc, F2163-A), TNF-α mouse ELISA test kit (shfksc, F2132-A), IL-1β mouse ELISA test kit (shfksc, F2040-B).

### 1.2 Main instruments

Rotary evaporator (Model: IKA RV10, made in Germany); MCO-18AIC constant temperature CO_2_ incubator (Model: CCL-240B-8, made in Japan); High performance liquid chromatograph (Model: LC-10A, infusion pump: LC-10ATVP, detector: SPD-20A, origin: Japan); Electric blast drying box (Model: WGLL-125BE, made in China); Full-wavelength automatic multi-functional enzyme marker (Model: MUItiskan GO, made in USA); Leica Fluorescent inverted microscope (Model: DMi8, made in Germany).

## 2 Data and methods

### 2.1 Extraction and identification of PFHF

#### 2.1.1 Ultrasonic extraction

We meticulously weighed 1 kilogram of PFH slices and thoroughly ground them into a fine powder. Subsequently, the powder was mixed with 10 liters of 90% ethanol at a ratio of 1:10. To fully extract its active ingredients, we employed an ultrasonic extraction method, repeating the process three times, each session lasting 40 minutes while maintaining a temperature of 55 °C to ensure extraction efficiency. After each ultrasonic extraction, we carefully filtered out the residue through fine gauze to obtain a clear extract. Following this, the three extracts were combined, and the ethanol was recovered using reduced pressure rotary evaporation. Finally, the resulting alcohol-free extract was further concentrated into a paste, with its optimal quality standard being that the ethanol concentration, as verified by three consecutive gas chromatography tests, remains below the detection limit of 0.1%.

#### 2.1.2 Resin preparation

The extract was subjected to elution and separation using D101 macroporous resin. The D101 macroporous resin was soaked in 95% ethanol for 12 hours, then rinsed with distilled water. The rinsing solution was collected every 100 mL, and ethanol residues were detected using gas chromatography. The chromatographic conditions were as follows: column: Agilent DB-WAX (30m×0.32mm×0.5μm); inlet temperature: 200 °C; detector temperature: 250 °C; temperature program: initial temperature 40 °C held for 3 min, increased to 200 °C at 10 °C/min, and held for 5 min; carrier gas: high-purity nitrogen, flow rate: 1.0 mL/min; injection volume: 1μL. Ethanol was considered completely removed when the peak area of ethanol was below the value corresponding to 0.1% of the standard curve in three consecutive tests. After standing for 12 hours, the distilled water was discharged.

#### 2.1.3 Elution process

The extract of “2.1.1” was then poured into the chromatographic column and eluted with 70% ethanol until nearly colorless. Subsequently, it was placed in a 100 °C water bath and boiled until it formed a thick, paste-like consistency, then placed in a brown bottle and set aside.

#### 2.1.4 HPLC conditions

Rutin content in PFHF was determined by high performance liquid chromatography (HPLC) to evaluate the quality of PFHF.

##### 2.1.4.1 Chromatographic conditions

The chromatography was performed on Nouryon Kromasil 100-5-C18 M05CLA25 (4.6mm×150mm, 5μm) column (1). Mobile phase is methanol with 1% acetic acid(volume ratio 30:70); Column temperature is 25 °C; the detection wavelength is 375nm (1). The flow rate is 1.0mL/min, and the sample size is 10μL.

##### 2.1.4.2 Preparation of test product solution

Take 0.05g of PFHF dry paste, weigh it accurately, and add 1mL of 75% (volume fraction, the same below) ethanol as the test solution (mass concentration: 0.05mg/mL), ultrasonic treatment (power: 500W, frequency: 40kHz) for 30min, take it out to room temperature, use 75% ethanol to make up the lost mass, shake well. The sample bottle was filtered by a 0.45μm microporous filter membrane, and a 75% ethanol-negative sample solution was prepared by the same method.

##### 2.1.4.3 Preparation of reference solution

Precision weighing rutin reference 0.1mg into 1mL EP tube as reference solution (its mass concentration is 0.1mg/mL).

##### 2.1.4.4 Specific test

The test product, the control product and the negative sample solution were determined according to the “2.1.4.1” chromatographic condition.

### 2.2 Network pharmacology

#### 2.2.1 Screening and determination of main components and action targets

Through literature research (2), the chemical components of PFHF were identified. Potential active components were screened on TCMSP (3) (https://old.tcmsp-e.com/tcmsp.php) based on ADME parameters (oral bioavailability OB ≥ 30%, drug-likeness DL ≥ 0.18). The predicted targets of these components were then retrieved from TCMSP, and the target names were normalized using the Uniprot KB module in the Uniprot database (4) (https://www.uniprot.org/) (unified to official gene symbols). For components not included in TCMSP, their Canonical Smiles numbers were obtained from PubChem (5) and input into the SwissTargetPrediction platform (6) (http://www.swisstargetprediction.ch/) for target prediction, with a probability threshold ≥ 0.1. A total of 44 active component targets of PFHF were ultimately obtained.

#### 2.2.2 Mapping of drug active ingredients-target network

Using Cytoscape3.7.1 software (http://www.cytoscape.org/) construction of pharmaceutical ingredients-target network. After the Network is formed, network topology parameters such as mediateness, connectivity and tightness are measured using the built-in Network Analyzer to obtain the more important active ingredients and targets and the relationship between them.

#### 2.2.3 Collection, screening and matching of inflammatory targets

In the GeneCards database (https://www.genecards.org/) (7), a search was conducted using “inflammation” as the keyword, and screening was performed based on a Relevance score ≥ 2 times the median (screening threshold: ≥11.28). In the OMIM database (https://www.omim.org) (8) and the DisGeNET database (https://www.disgenet.org) (9), screening was carried out according to a Score ≥ 1 time the median (screening threshold: ≥0.1). Drug targets related to inflammation were searched for in the DrugBank database (https://go.drugbank.com/) (10). The inflammation targets retrieved from the DrugBank database also required normalization of the collected disease target names using UniprotKB in the Uniprot database (https://www.uniprot.org/) (4), unifying them into official gene symbols (Gene Symbol). Ultimately, inflammation-related targets were obtained. Then, the targets corresponding to the screened active ingredients of PFHF and the inflammation-related targets were matched by creating a Venn diagram (http://bioinformatics.psb.ugent.be/webtools/Venn/), and the intersection of the two was taken to obtain the potential target genes of PFHF for treating inflammation.

#### 2.2.4 Construction of protein-protein interaction network and screening of core targets

PPI network map was constructed based on STRING database (https://string-db.org/) based on the previously analyzed intersection target genes of PFHF active component targets and inflammatory disease targets, screened (the standard was interactionscore>0.4), and excluded isolated proteins without interaction. PPI network map was combined with Cytoscape3.7.1 for visualization and beautification. PPI network map of differentially expressed genes was selected based on the interaction index of proteins. Topological analysis of PPI networks is performed using the CytoNAC plug-in in Cytoscape3.7.1 software. The top 50% of nodes are selected as the screening criteria, and topological analysis is performed on point centrality, intermediate centrality, and proximity centrality in order to screen more important target genes, namely: The core target of PFHF for treating inflammation.

#### 2.2.5 GO and KEGG signaling pathway analysis

Based on Metascape (https://metascape.org/gp/index.html#/main/step1), GO functional enrichment analysis and KEGG pathway enrichment analysis were conducted with the support of Weishengxin (https://www.bioinformatics.com.cn). (The enrichment analysis employed the hypergeometric test algorithm and utilized the Benjamini-Hochberg (BH) method for false discovery rate (FDR) correction. A corrected P-value (i.e., FDR value) < 0.05 was used as the significance threshold.) According to the criteria of P < 0.05 and ascending order, the top 10 GO enrichment entries were selected. After converting the P-values to -LogP, a three-in-one visualized bar chart for biological processes (BP), cellular components (CC), and molecular functions (MF) was plotted. Meanwhile, for the KEGG enrichment analysis, the P-values < 0.05 were arranged in ascending order, and the top 20 KEGG signaling pathways were selected to plot a bubble chart. Finally, a “drug component-intersection target-signaling pathway” network diagram was constructed using Cytoscape 3.7.1.

### 2.3 Molecular docking and dynamics simulation of components and targets

Molecular docking was performed using the targets of the top five key active ingredients, ranked by Degree value, from the PPI network of intersecting genes involved in PFHF-mediated inflammation treatment. First, high-resolution crystal structures of the core targets were downloaded from the RCSB PDB database (http://www.rcsb.org/).For all receptor proteins, conformations complexed with native ligands or known inhibitors were selected.

The structural formula of the studied component (rutin) was retrieved from TCMSP, downloaded in mol2 format, and used as the docking ligand. The AutoDockTools 1.5.6 software was employed for preprocessing both the receptor and ligand. Receptor protein preparation included the removal of all water molecules, elimination of native ligands, addition of polar hydrogen atoms, and calculation of Gasteiger charges. Ligand preparation involved adding nonpolar hydrogen atoms, defining rotatable bonds, and calculating Gasteiger charges. The protonation states of all molecules were determined at pH 7.4. The center coordinates of the docking grid box were set to cover the binding pocket of the native ligand. Subsequently, STRING was used to retrieve the corresponding component’s PDB, and the PDB database (http://www.rcsb.org/) was linked to PDB RCSB (https://www.rcsb.org/structure/5n10) to search for and collect suitable crystal structures (with resolution < 3 Å and complete pocket structures) as receptor proteins. Using PyMOL software, the receptor proteins were prepared by removing water molecules and ligands before mutual docking. The Autodock software (11) was then used for docking procedures, including hydrogen addition and charge calculation, before initiating docking with the aforementioned structures. AutoDock Vina 1.1.2 was employed for semi-flexible docking calculations, with the exhaustiveness parameter set to 8 and other parameters kept at default values. The binding energy was obtained, with lower binding energies indicating a tighter match between the docked ligand and receptor.

To further validate the binding stability and dynamic interaction patterns between the key active ingredient (rutin) and the core targets in molecular docking, this study specifically performed molecular dynamics(MD) simulations on the rutin–TNF and rutin–INS complex systems, which exhibited favorable binding energy in the molecular docking results. All-atom MD simulations were performed for 100 ns using GROMACS 2024.5 (https://www.gromacs.org) (12). The simulations utilized the amber14sb force field and the TIP3P water model under truncated octahedron periodic boundary conditions (13). The system charge was neutralized by adding Na+/Cl- ions. Electrostatic interactions were calculated using the Particle Mesh Ewald (PME) method, with a cutoff distance of 10 Å for van der Waals interactions. Bonds involving hydrogen atoms were constrained using the LINCS algorithm (14). Energy minimization was carried out using the steepest descent method until the maximum force fell below 10.0 kJ/mol. During the pre-equilibrium phase, the velocity Verlet integrator was employed, with temperature coupling controlled by the V-rescale method and pressure coupling achieved using the C-rescale method. An initial equilibration of 100 ps was conducted to stabilize the system at 298.15 K and 1 bar. Production MD simulations were run with a time step of 2 fs for a total duration of 100 ns. Trajectory frames were recorded every 100 ps, resulting in a total of 1000 conformational snapshots. The built-in tools of GROMACS were used to calculate the root mean square deviation (RMSD), root mean square fluctuation (RMSF), radius of gyration (Rg), and number of hydrogen bonds (Hbond).

### 2.4 Association between core targets and KEGG pathways

The most significantly enriched KEGG signaling pathways analyzed in section “2.2.5” were obtained from the KEGG online website (https://www.kegg.jp). A network diagram encompassing all core targets identified in section “2.2.4” was selected to visually demonstrate the inflammatory mechanism associations between core targets and KEGG pathways.

### 2.5 Cell validation experiment

#### 2.5.1 Cell culture

RAW264.7 cells were cultured in DMEM medium containing 10% fetal bovine serum, 1% penicillin and streptomycin, and in a cell incubator at 37°C and 5% CO_2_ with saturated humidity. When the cells reached 80% to 90%, they were passed by 1:2 to 1:4, about 3 to 5 days, and cells in the logarithmic growth stage were selected for reserve and follow-up experiments.

#### 2.5.2 Cell proliferation experiment

RAW264.7 cells of logarithmic growth stage were inoculated into 96-well plates at a density of 1×10^5^/ml with 100μl per well and cultured at 37°C for 24 hours. RAW264.7 was divided into the following 10 groups and given the following 10 different interventions: Just CCK8 solution, 7.81μg/mL PFHF and CCK8 solution, 15.63μg/mL PFHF and CCK8 solution, 31.25μg/mL PFHF and CCK8 solution, 62.5μg/mL PFHF and CCK8 solution, 125μg/mL PFHF and CCK8 solution, 250μg/mL PFHF and CCK8 solution, 500μg/mL PFHF and CCK8 solution,1000 μg/mL PFHF and CCK8 solution and Just PBS not cells and CCK8 solution. The group adding only CCK8 solution was the normal control group, the group adding PFHF was the experimental group, the group adding only PBS but no cells and CCK8 solution was the blank zeroing group. Each group has 6 multiple holes. After all cells were treated for 24 hours and 48 hours, 5μL CCK-8 reagent was added to each well, and the cells were mixed and incubated for 1 hour. Optical Density (OD) was measured at 450nm. Taking the blank group as the zeroing hole, the cell survival rate of each group was {(OD total flavone-OD zeroing group)/(OD control -OD zeroing group)}×100%.

#### 2.5.3 Establishment of inflammation model of RAW264.7 cells *in vitro* and administration intervention

RAW264.7 cells from the logarithmic growth stage were inoculated into 6-well plates at a density of 10×10^4^ cells/ml, with 2mL per well, and incubated in incubators at 37°C, 60% humidity and 5%CO_2_ for 24 hours. The specific groups were as follows: blank group, LPS model group, Prednisolone acetate group, PFHF L group and PFHF -H group. 1 mL complete medium was added to the blank group. The other four groups were added 1 mL LPS complete medium solution containing 1 μg/mL. After incubation at 37°C for 6 hours, each administration group was added: blank group: no treatment was done; LPS model group: no treatment was done. In prednisolone acetate group, the supernatant was discarded and 1mL of 10 nmol/mL prednisolone acetate solution was added. PFHF L group: Discard the supernatant and add 1mL of 27.5 μg/mL PFHF solution; PFHF H group: Discard the supernatant and add 1mL of 110μg/mL PFHF solution(The selection of high and low doses was based on the IC50 values determined by the CCK-8 assay: the high dose was slightly lower than the 24-hour IC50 but higher than the 48-hour IC50, aiming to balance cell survival with the observation of anti-inflammatory effects; the low dose was significantly lower than the IC50 values at both time points, ensuring specific evaluation of anti-inflammatory activity rather than cytotoxicity. The combination of these doses allows for a comprehensive analysis of the dose-dependent anti-inflammatory mechanism of PFHF). The culture was continued at 37°C for 18 hours.

#### 2.5.4 Determination of IL-6, TNF-α and IL-1β in cell culture medium

RAW264.7 cells were modeled according to the method in item 2.4.3 and 24 hours after administration, cells and culture medium of each group were collected to prepare the freeze-thaw solution of RAW264.7 cells. The contents of IL-6, TNF-α and IL-1β were determined according to the instructions of the ELISA kit.

#### 2.5.5 Immunofluorescence

The RAW264.7 cells were modulated according to the method in item 2.4.3 and 24 hours after administration, the liquid in the pores was discarded, each well was cleaned with PBS, and then fixed with 4% paraformaldehyde for 15 minutes, and cleaned with PBS for 3 times. The expression of INS, CASP3, iNOS and Cox-2 protein in each group was detected by immunofluorescence staining. The first antibody was incubated at 4°C overnight (the dilution of INS, CASP3, iNOS and Cox-2 were all 1:500), and the second antibody was incubated for 90 minutes under light protection. The nuclei were re-dyed by 4,6-diamino-2-phenyl indole(DAPI), observed under fluorescence microscope and photographed. Three fields of view were taken in each group.

### 2.6 Statistical analysis

Data were analyzed using GraphPad Prism software (version 8.0.2). Quantitative data are presented as mean ± standard deviation. Differences between two groups were assessed using independent samples t-test, while comparisons across multiple groups were performed via one-way analysis of variance (ANOVA), followed by Tukey’s *post hoc* test for pairwise comparisons. A P-value <0.05 was considered statistically significant.

## 3 Results

### 3.1 Determination results of rutin content in PFHF

**Figure 1:**
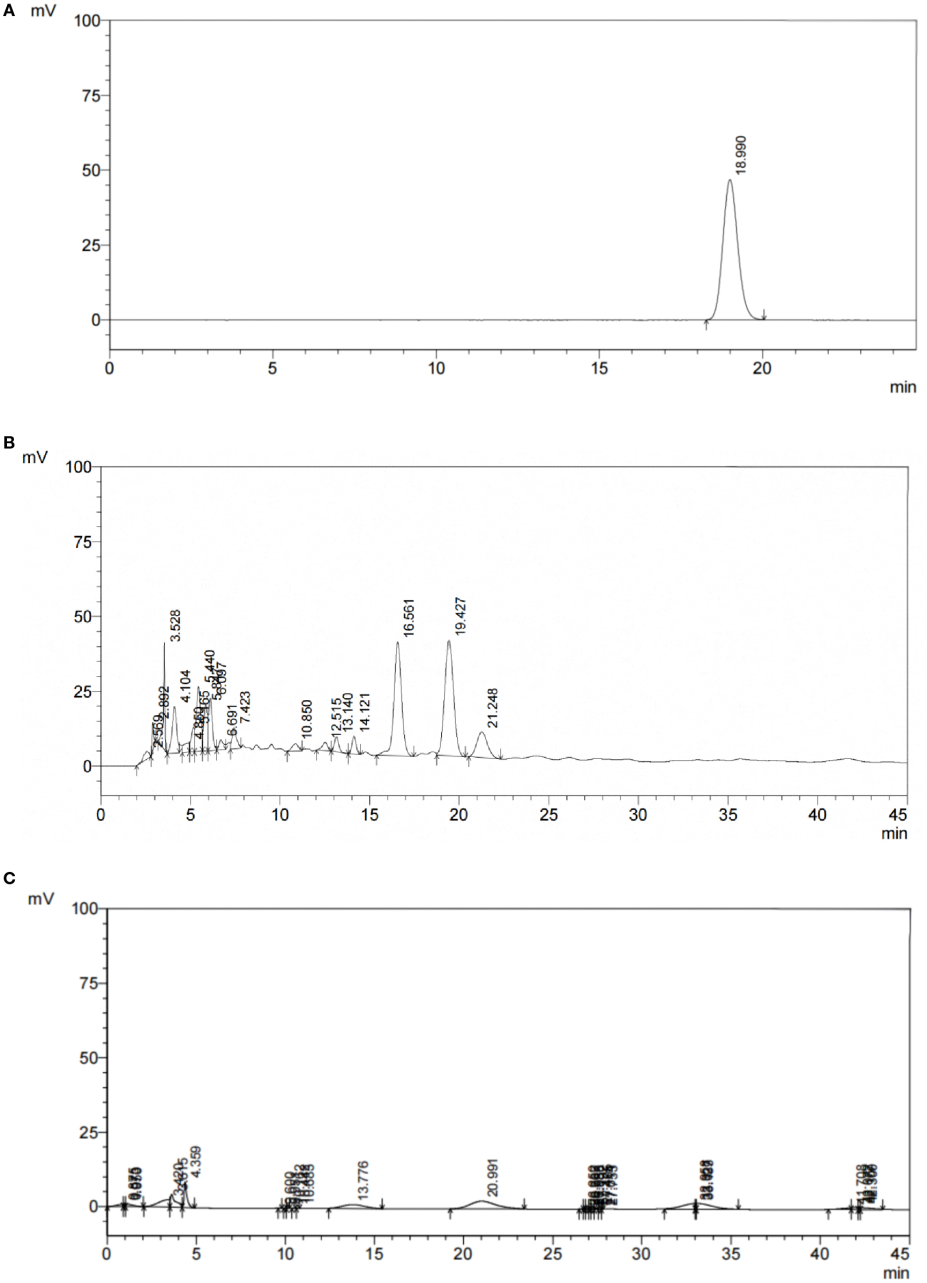
HPLC chromatogram of PFHF, **(A)** Reference Standard; **(B)** Test Solution; **(C)** Negative Control.

HPLC results showed that in the total flavonoid detection area, the sample had a peak (see **Figure 1**), indicating that the sample was a water lily, which provided a guarantee for the subsequent drug administration quality of this experiment. Moreover, the theoretical plate number of total flavonoids rutin is n=5.54 (t_R_/W_h/2_) 2, where: n is the theoretical plate number; t_R_ is the retention time; W_h/2_ is half peak width; The separation degree R = 2 (t_R2_-t_R1_)/(W_2_ +W_1_), where t_R2_ and W_2_ are the retention time and peak width of the last of the two adjacent chromatographic peaks respectively, and t_R1_ and W_1_ are the retention time and peak width of the first of the two adjacent chromatographic peaks respectively. The contents of rutin in the sample are shown in **Table 1**. The chromatographic conditions can have a good separation degree of rutin in the test product, the separation degree is greater than 1.5, and the baseline separation is reached.

**Table 1:** Theoretical plate number and separation degree.

Determination solution	t_R_/min	n	R	Area of Ruding Peak	Sample content mg/ml
Sample for test	18.990	8308	3.423	1320716	0.09
Reference substance	19.427	7253	–	1480764	0.1
Negative control	–	–	–	–	–

### 3.2 Active ingredients of PFHF and their targets

**Figure 2:**
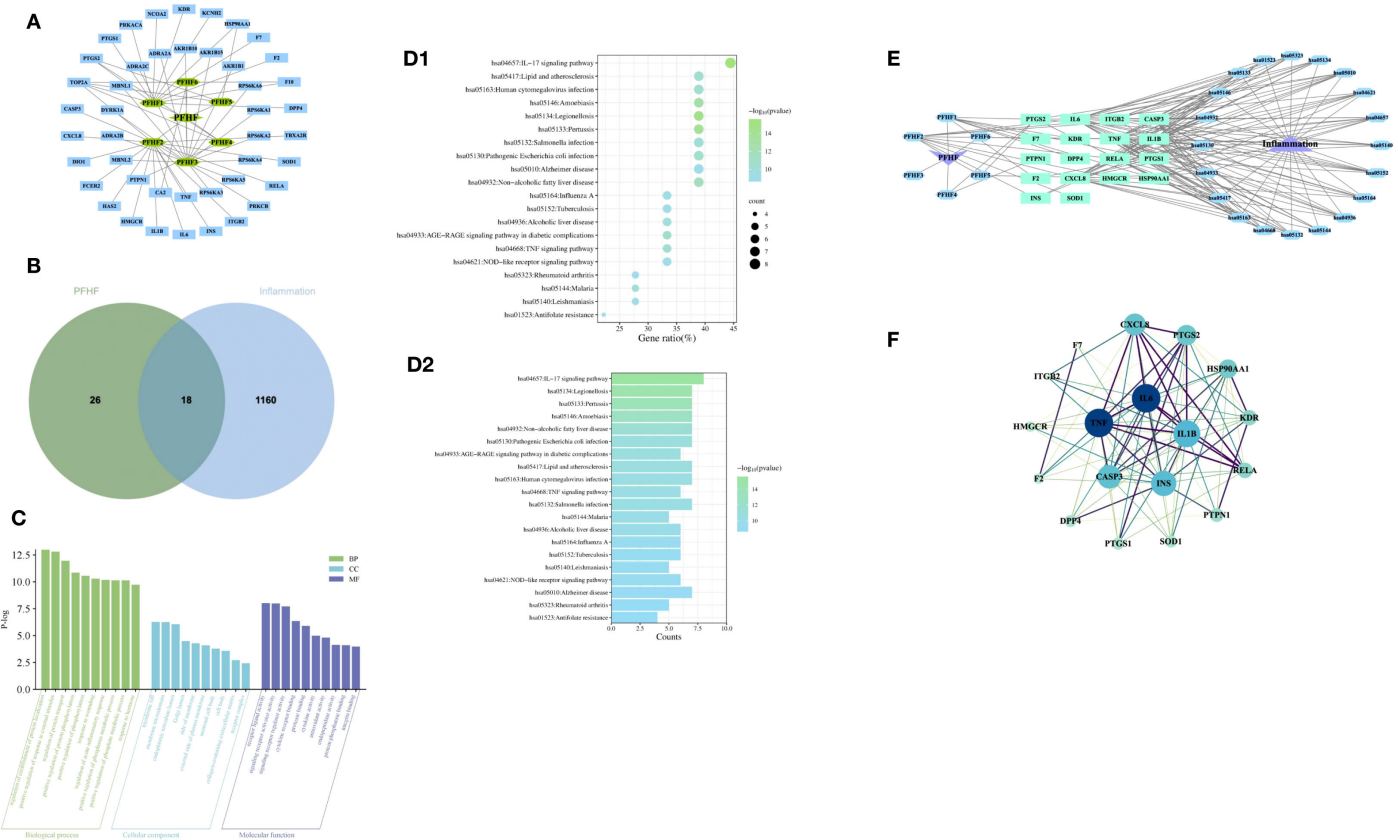
**(A)** PFHF component - target network; **(B)** Venn map of genes associated with PFHF treatment of inflammation; **(C)** GO functional analysis; **(D1)** Bubble map of KEGG enrichment analysis, the x-axis (Gene Ratio, %) represents the ratio of the number of PFHF core targets enriched in a given pathway to the total number of all core targets; **(D2)** Histogram of KEGG enrichment analysis; **(E)** PFHF component-target-pathway network; **(F)** PPI network map and core targets of inflammatory targets treated by PFHF. PFHF1, porphyrin; PFHF2, quercetin 3-O-β-D-glucoside; PFHF3, quercetin-3-O-beta-xylopyranoside; PFHF4, quercetin-3-O-α-L-arabinopyranoside; PFHF5, isorhamnein-3-O-β-glucoside; PFHF6, Rutin. PFHF, total flavonoids of *Polygala fallax* Hemsl.; hsa04657,IL-17 signaling pathway;hsa05134,Legionellosis;hsa05133,Pertussis;hsa05146,Amoebiasis;hsa04932,Non-alcoholic fatty liver disease;hsa05130,Pathogenic Escherichia coli infection;hsa04933,AGE-RAGE signaling pathway in diabetic complications;hsa05417,Lipid and atherosclerosis;hsa05163,Human cytomegalovirus infection;hsa04668,TNF signaling pathway;hsa05132,Salmonella infection;hsa05144,Malaria;hsa04936,Alcoholic liver disease;hsa05164,Influenza A;hsa05152,Tuberculosis;hsa05140:Leishmaniasis;hsa04621:NOD-like receptor signaling pathway;hsa05010:Alzheimer disease;hsa05323:Rheumatoid arthritis;hsa01523:Antifolate resistance.

A total of 6 PFHF components were obtained, as shown in **Table 2**. After the above chemical components were matched with action targets, 44 active ingredient action targets were obtained by combining and deleting duplicate values. The network of active ingredient-target was shown in **Figure 2A**.

**Table 2:** Components of PFHF.

ID	Molecule name	Chemical formula
MOL000561	Kaempferol 3-O-beta-D-glucopyranoside	C_21_H_20_O_11_
CAS56316-75-7	Quercetin-3-O-(6''-galloyl)-β-D-glucoside	C_21_H_20_O_12_
MOL004354	Quercetin 3-O-beta-D-xylopyranoside	C_20_H_18_O_11_
MOL013069	Quercetin 3-O-α-L-arabinopyranoside	C_20_H_18_O_11_
MOL012595	Isorhamnetin-3-O-glucoside	C_22_H_22_O_12_
MOL000415	Rutin	C_27_H_30_O_16_

### 3.3 Core targets of PFHF in the treatment of inflammation

The intersection of PFHF component target genes and inflammation target genes was taken, resulting in 18 target genes for PFHF in treating inflammation, which are: DPP4, F7, CXCL8, TNF, F2, PTGS2, ITGB2, IL6, HSP90AA1, CASP3, HMGCR, KDR, INS, PTPN1, SOD1, RELA, IL1B, and PTGS1, as shown in **Figure 2B**.

### 3.4 GO function analysis of the core target of PFHF in the treatment of inflammation

The GO function analysis of the core targets of PFHF for the treatment of inflammation obtained 477 items with significant differences, among which the top 10 BP were mainly involved in the positive regulation of the establishment of protein localization, the positive regulation of the response to external stimuli, and the regulation of protein transport. CC mainly involves membrane raft, membrane microregion, endoplasmic network cavity and so on. MF mainly involves receptor ligand activity, signal receptor activator activity, and signal receptor regulatory activity, as shown in **Figure 2C**.

### 3.5 PFHF active ingredients treat KEGG signaling pathway at the core target of inflammation

There are 74 signaling pathways for PFHF active ingredients to treat core targets of inflammation, The top 20 significantly differentially enriched signaling pathways include the IL-17 signaling pathway, Legionellosis, Pertussis, Amoebiasis, Non-alcoholic fatty liver disease, Pathogenic Escherichia coli infection, and AGE-RAGE signaling pathway in diabetic complications, Lipid and atherosclerosis, Human cytomegalovirus infection, and TNF signaling pathway, Salmonella infection, Malaria, Alcoholic liver disease, Influenza A, Tuberculosis, Leishmaniasis, NOD-like receptor signaling pathway, Alzheimer’s disease, Rheumatoid arthritis, and Antifolate resistance are shown in **Figures 2D1, D2**, suggesting that PFHF components may play a role in treating inflammation by acting on the signaling pathways where these targets are located. Finally, the PFHF component-target-pathway network was constructed using Cytoscape3.7.1, as shown in **Figure 2E**.

### 3.6 PPI network construction and core target screening

The therapeutic targets of PFHF active components for inflammation include DPP4, F7, CXCL8, TNF, F2, PTGS2, ITGB2, IL6, HSP90AA1, CASP3, HMGCR, KDR, INS, PTPN1, SOD1, RELA and I L1B, PTGS1, after using Cytoscape beautification as shown in **Figure 2F**, according to the interactionscore > 0.4 filter and eliminate isolated protein, without interaction, according to the Degree value sorting, The top 5 gene targets of Degree value IL6, TNF, IL-1β, INS and CASP3 were selected as the core targets of PFHF in the treatment of inflammation for subsequent molecular docking verification.

### 3.7 Molecular docking of important components and targets of PFHF for the treatment of inflammation

Core targets IL6, TNF, IL-1β, INS, CASP3 and their corresponding compounds (see **Table 3**), which ranked among the top 5 in the PPI network of intersection genes used by PFHF to treat inflammation, were selected for molecular docking.

**Table 3:** Core targets of inflammation treated by PFHF.

Sort	Core target	Mark	Corresponding compound
1	IL6	17	Rutin
2	TNF	17	Rutin
3	IL-1β	16	Rutin
4	INS	15	Rutin
5	CASP3	14	Rutin

IL-6 (PDB ID: 4CNI, resolution: 1.90 Å), TNF (PDB ID: 3L0V, resolution: 2.10 Å), IL-1β (PDB ID: 5R8Q, resolution: 2.60 Å), INS (PDB ID: 5E7W, resolution: 2.30 Å), and CASP3 (PDB ID: 6BFO, resolution: 2.36 Å).Specific dimensions and center coordinates as follows: IL-6 (size: 40×40×40 Å³, center coordinates: x=10.512, y=53.981, z=35.218), TNF (size: 42×42×42 Å³, center coordinates: x=35.661, y=35.432, z=35.211), IL-1β (size: 38×38×38 Å³, center coordinates: x=12.654, y=45.321, z=23.876), INS (size: 36×36×36 Å³, center coordinates: x=15.432, y=32.123, z=10.987), and CASP3 (size: 40×40×40 Å³, center coordinates: x=25.678, y=15.432, z=12.345). The grid spacing was set to 0.375 Å.

**Figure 3:**
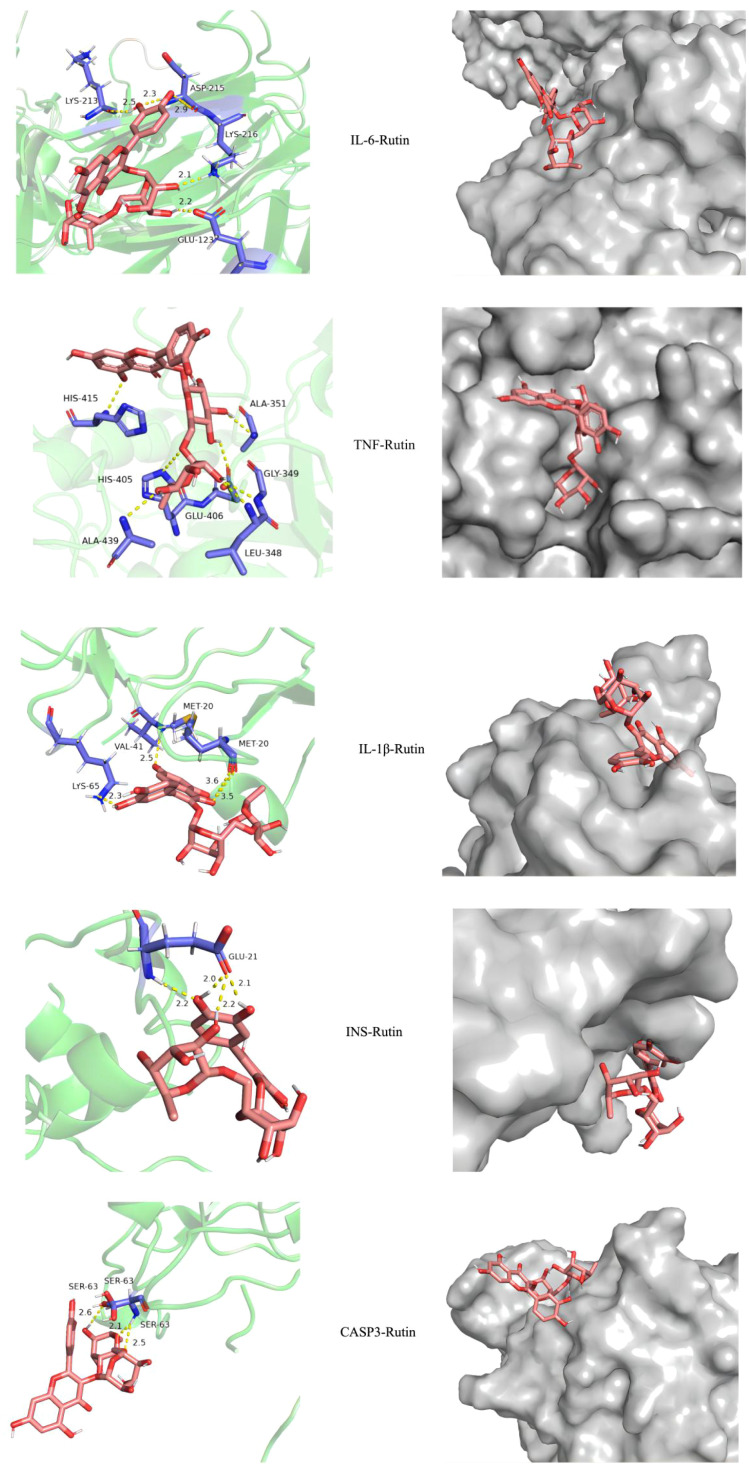
Visualization of molecular docking results.

Molecular docking results showed that the docking binding energy was all less than 0, indicating that all of them could spontaneously bind to target genes. The smaller the binding energy was, the easier it was to bind, and the smaller the binding energy was, the more stable the binding energy between ligand and target was, as shown in **Table 4**. The docking compound results derived from Autodock4 were imported into Pymol, and Pymol was used to perform molecular three-dimensional display of the compound. Part of the docking results are shown in **Figure 3**.

**Table 4:** Molecular docking binding energy of core targets.

Core target	Active ingredient	Binding energy(kcal/mol)	PDB id
IL-6	Rutin	-0.21	4CNI
TNF	Rutin	-8.70	3L0V
IL-1β	Rutin	-1.62	5R8Q
INS	Rutin	-1.66	5E7W
CASP3	Rutin	-0.60	6BFO

**Figure 4:**
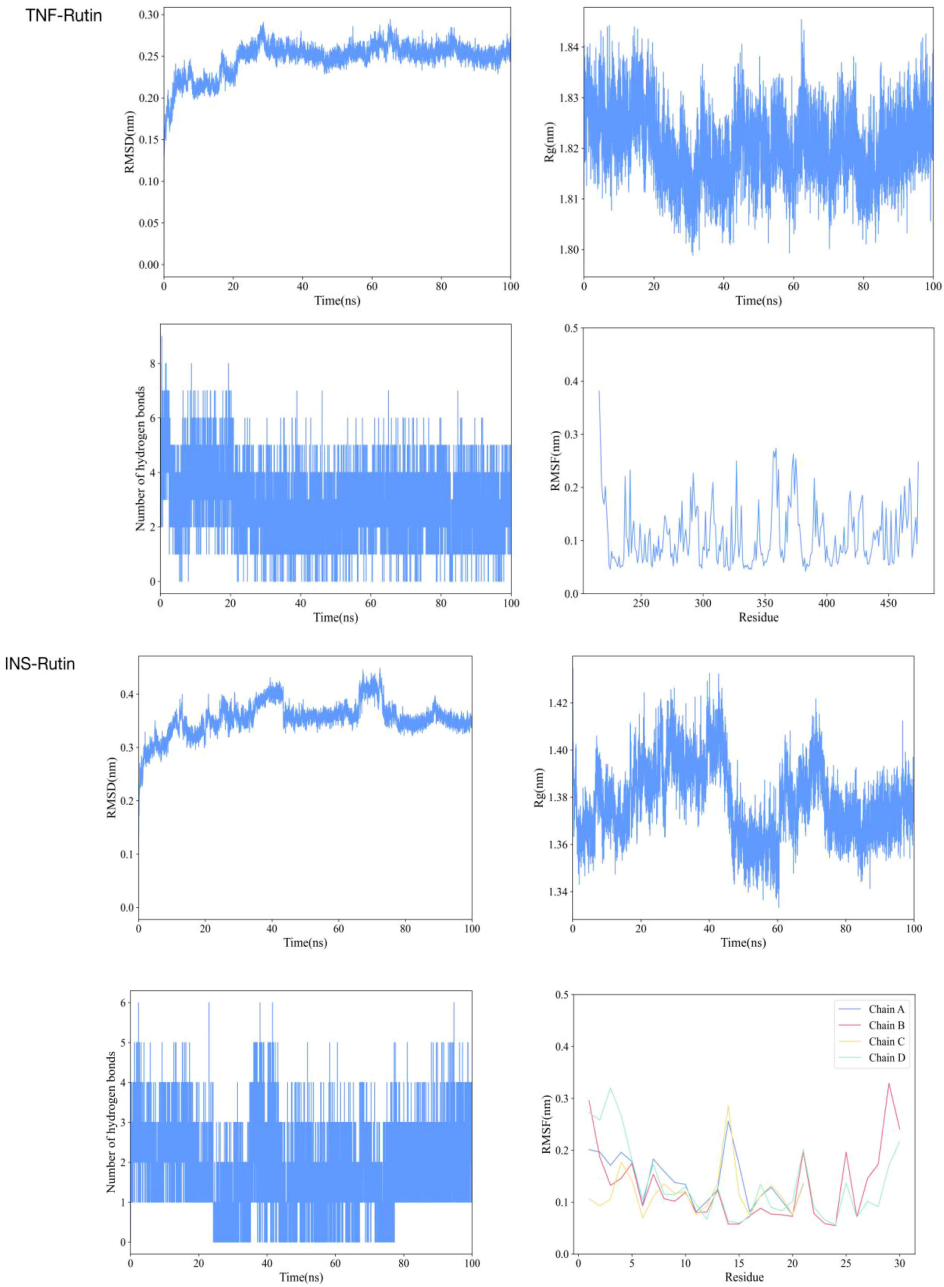
Molecular dynamics simulation results of TNF and INS with Rutin, respectively.

Based on the provided molecular dynamics simulation data, the interaction results between INS/TNF and Rutin reveal the following: Throughout the simulation, the RMSD values of INS show a fluctuating upward trend and eventually stabilize within the range of approximately 0.32 to 0.38 nm, indicating that the structure of the INS-Rutin complex gradually reaches a stable state. In contrast, the RMSD values of TNF remain relatively low and stable, maintained between 0.16 and 0.26 nm, demonstrating greater structural rigidity after binding with Rutin. Rg analysis indicates that the Rg values of the INS-Rutin complex slightly increase in the later stages of the simulation, suggesting a mild structural expansion of the system. Conversely, the Rg values of the TNF-Rutin complex remain stable, indicating a compact and stable structure. Hydrogen bond analysis shows that a dynamically changing hydrogen bond network forms between INS and Rutin, with an average of about 1.8 hydrogen bonds, contributing to the stability of the complex. Although hydrogen bonding interactions exist between TNF and Rutin, they are fewer in number but more stable. RMSF analysis reveals differences in residue fluctuations among chains A, B, C, and D of INS, with certain regions such as residue 14 in chain A and residue 29 in chain B exhibiting higher RMSF values, indicating greater flexibility in these areas. In contrast, the residue RMSF values of TNF are generally low, suggesting overall structural stability after binding with Rutin. Results are shown in **Figure 4**.

### 3.8 Visualization of core target-pathway associations

**Figure 5:**
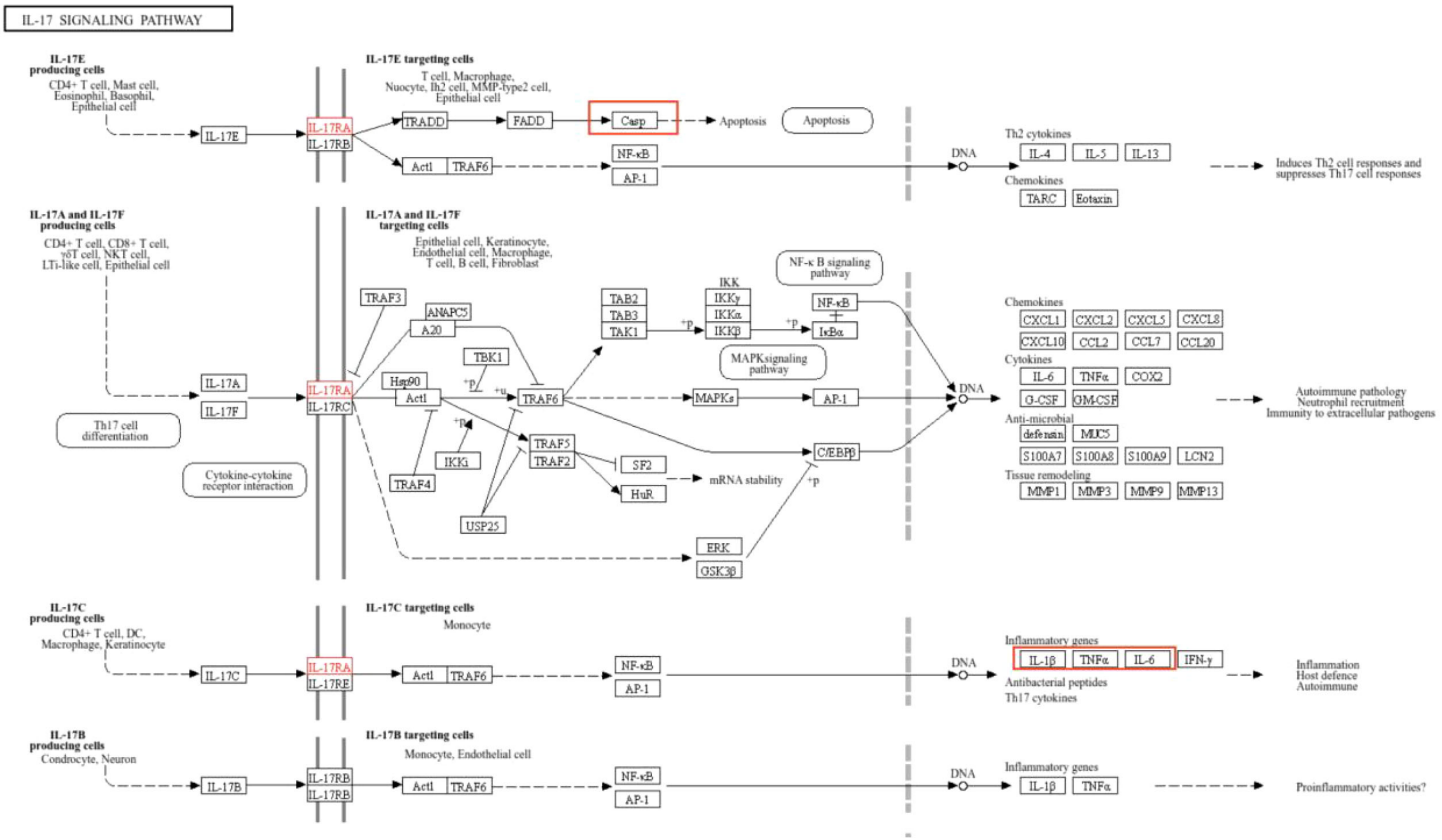
Interaction network of IL-17 signaling pathway with core targets.

The IL-17 signaling pathway (k05164) was obtained from the KEGG database. Upon selecting map04657, it is evident that IL-6, TNF, and IL-1β form a highly interconnected inflammatory regulatory hub within the IL-17 signaling pathway. While CASP3 demonstrates close association with apoptotic processes, insulin (INS) is not directly visualized in this pathway map (**Figure 5**).

### 3.9 Results of cell proliferation experiment

**Figure 6:**
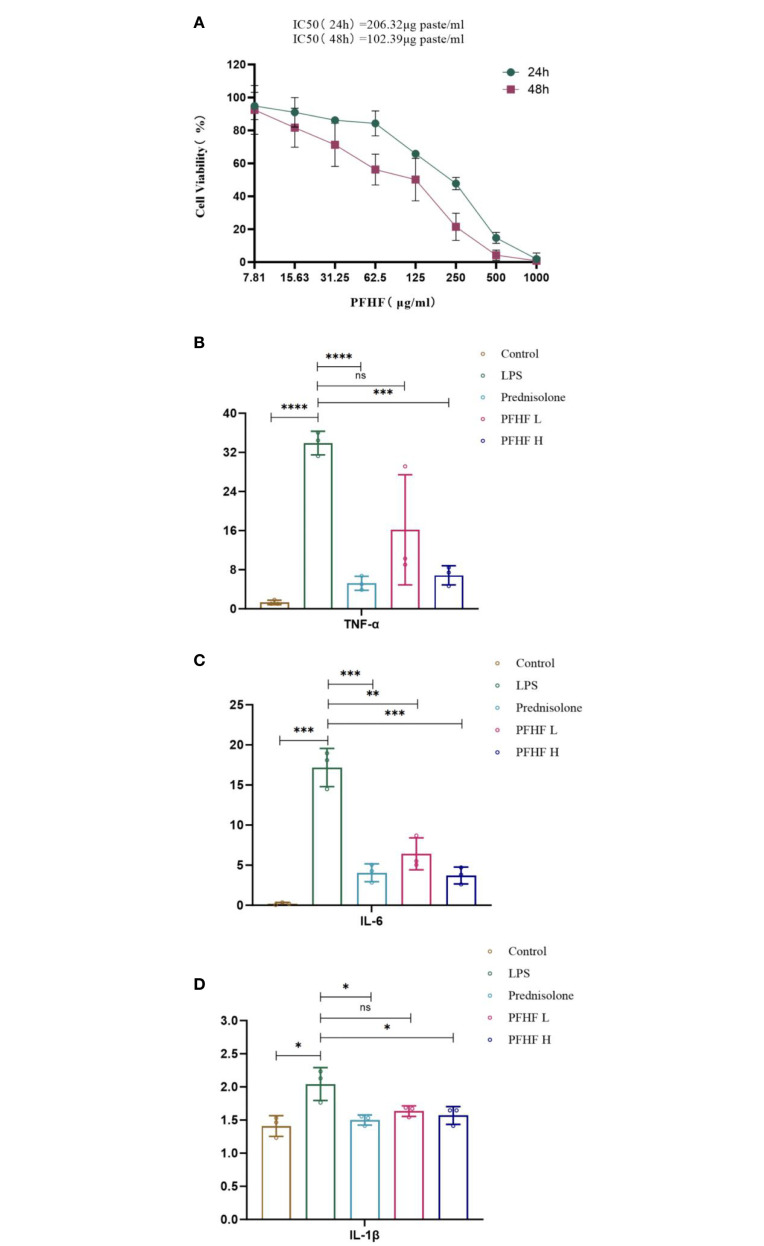
**(A)** Effects of PFHF on proliferation of RAW264.7 cells; **(B–D)** Effects of PFHF on TNF-α, IL-6 and IL-1β of RAW264.7 cells. *P<0.05, **P<0.01, ***P<0.001, **** P<0.0001. Contral, normal control group; LPS, inflammation model group; Prednisolone, Prednisolone group; PFHF L, low dose group of PFHF; PFHF H, high dose group of PFHF. ns, Not significant.

The results of CCK-8 showed that the survival rate of RAW264.7 cells decreased with the increase of drug concentration after different concentrations of total flavone treated RAW264.7 cells for 24 and 48 hours, respectively, and the proliferation inhibition effect of PFHF on RAW264.7 was time-dose-dependent (**Figure 6A**). The IC50 (24 hours) was 206.32µg paste/mL, and the IC50 (48 hours) was 102.39µg paste/mL.

### 3.10 Effects of total flavones of *Psammoma japonica* on the contents of inflammatory factors TNF-α, IL-6 and IL-1β in RAW264.7 cells induced by LPS

The experimental results showed that compared with the blank control group, the levels of TNF-α, IL-6 and IL-1β in the freeze-thaw fluid of inflammatory model cells were significantly increased, and the differences were statistically significant (*P<0.05* Note the italics of the statistical value); Compared with the inflammatory model group, the levels of TNF-α, IL-6 and IL-1β of prednisolone acetate and total flavonoids in low-dose and high-dose groups were decreased (all *P<0.05*, except for IL-6 and IL-1β levels in low-dose group). See **Figures 6B–D**.

### 3.11 Effects of total flavones of P. flavones on the expression of INS, CASP3, iNOS and Cox-2 protein in LPS-induced RAW264.7 cells

**Figure 7:**
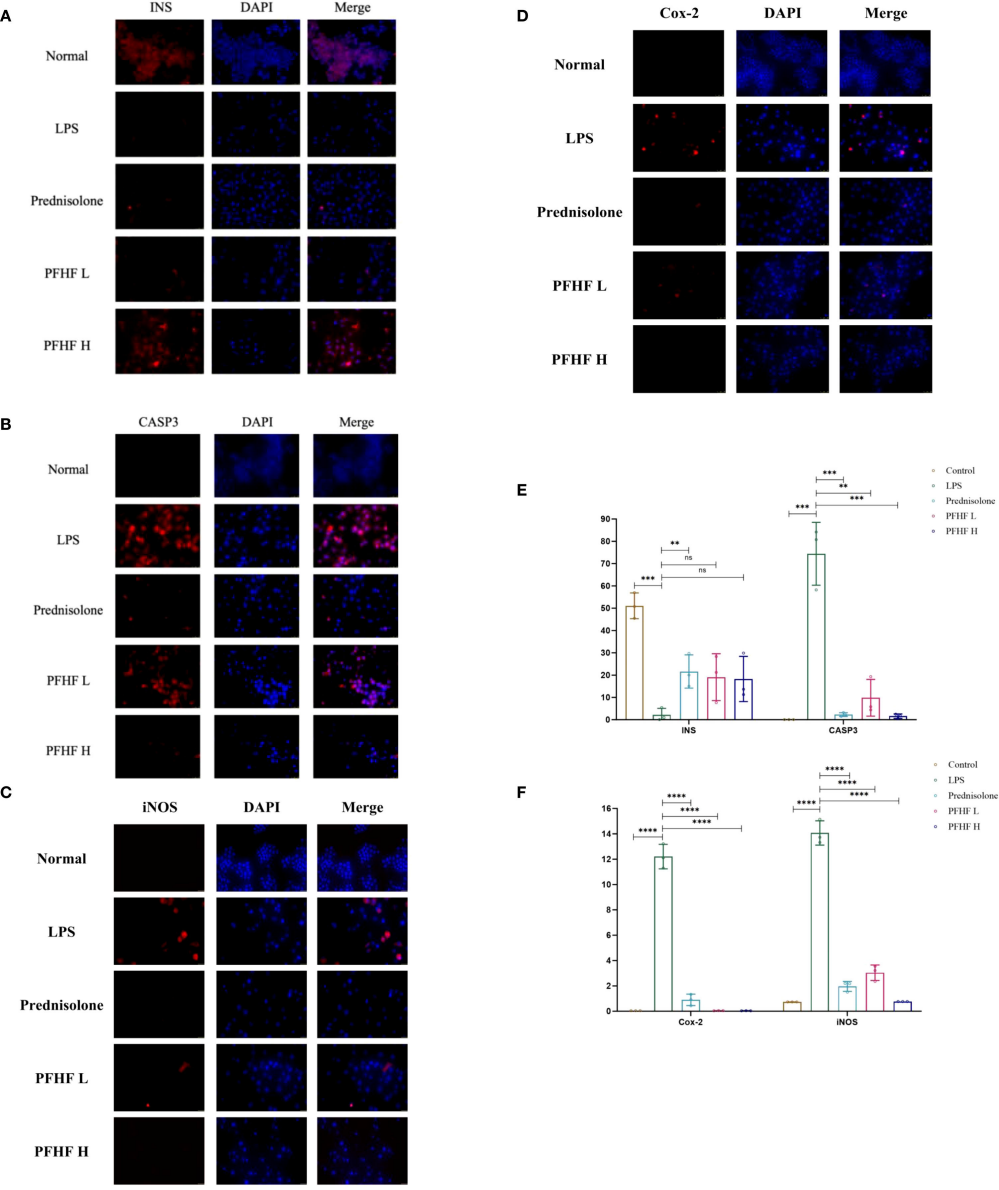
**(A)** Expression of INS protein in RAW264.7 cells induced by PFHF (×400, scale: 25μm); **(B)** Expression of CASP3 protein in RAW264.7 cells induced by PFHF (×400, scale: 25μm); **(C)** Expression of Cox-2 protein in RAW264.7 cells induced by PFHF (×400, scale: 25μm); **(D)** iNOS protein expression in RAW264.7 cells induced by PFHF (×400, scale: 100μm); **(E)** Expression of INS and CASP3 protein in RAW264.7 cells induced by PFHF (n=3). **(F)** Expression of Cox-2 and iNOS protein in RAW264.7 cells induced by PFHF (n=3). ns, Not significant. **P<0.01, ****P< 0.0001.

In **Figures 7A–D**, the blue fluorescence is the nuclear DAPI label, and the red fluorescence is the expression of the target protein.

The immunofluorescence expression of INS protein was stronger in the normal group. Compared with the normal group, the immunofluorescence expression of INS protein in inflammatory model and PFHF group was decreased. There was a significant difference between the normal group and the inflammatory model (*P < 0.0001*), while there was no significant difference between the inflammatory model group and the low-dose and high-dose PFHF groups. However, the fluorescence distribution in the PFHF group was more than that in the inflammatory model group through observation and calculation (*n=3*). See **Figure 7E**.

The immunofluorescence expression of CASP3 protein was weak in the normal group. Compared with the normal group, the immunofluorescence expression of CASP3 protein in inflammatory model and PFHF group was enhanced. There were significant differences in immunofluorescence expression of CASP3 protein between the normal group and the inflammatory model (*P < 0.0001*). The immunofluorescence expression of CASP3 protein in the inflammation model group also demonstrates significant differences compared to that in the prednisolone group, the low-dose PFHF group, and the high-dose PFHF group. (*P < 0.0001*). Moreover, the immunofluorescence expression of the high-dose PFHF group was lower than that of the prednisolone group, although the difference was not obvious, the fluorescence distribution could be reduced by calculation and observation (*n=3*). See **Figure 7E**.

The immunofluorescence expression of iNOS and Cox-2 protein in normal group was weak. The immunofluorescence expression of iNOS and Cox-2 protein in inflammatory model and PFHF group was enhanced compared with normal group. There were significant differences between the normal group and the inflammatory model (*P < 0.0001*), and the immunofluorescence expression of iNOS and Cox-2 protein between the inflammatory model group and the prednisolone group, as well as between the low and high dose groups of PFHF (*P < 0.0001*). See **Figure 7E**.

In response to the aforementioned data, we have also conducted a quantitative analysis, with the results presented in **Table 5**.

**Table 5:** Quantitative results of fluorescence intensity for INS, CASP3, iNOS, and Cox-2.

Group\Gene	INS	CASP3	Cox-2	iNOS
Control	51.1 ± 4.7	0.04 ± 0	0.7 ± 0	0.04 ± 0
LPS	2.2 ± 2.4	74.3 ± 11.5	14.1 ± 0.8	12.2 ± 0.8
Prednisolone	13.3 ± 1.6	2.3 ± 0.7	2.0 ± 0.3	0.9 ± 0.4
PFHF L	11.7 ± 6.7	9.8 ± 6.7	3.0 ± 0.5	0.04 ± 0
PFHF H	18.3 ± 8.3	1.6 ± 0.8	0.0008 ± 1.8	0.04 ± 0

## 4 Discussion

Inflammation can cause oxidative stress and generate oxidants such as reactive oxygen species and active nitrogen, which damage nucleic acids, proteins and lipids and induce gene mutation and tumor transformation (15). Zhou et al (16)found that the expression of genes TINAG, DDC, SPDEF and APOBEC1 involved in acute or chronic pancreatitis were significantly increased in pancreatic cancer, and they could be used as predictors of new pancreatic adenocarcinoma. Therefore, the treatment of inflammation should arouse the attention of the medical community in the early stage to prevent the deterioration of the condition. It has been widely used as folk medicine in Zhuang and Yao ethnic living areas, and has achieved remarkable curative effect on post-disease diseases such as body deficiency, waist and knee soreness, acute and chronic hepatitis, but its molecular mechanism is still unclear. Therefore, the mechanism of action between the two was analyzed by means of network pharmacology and bioinformatics.

Through the retrieval of drug target genes and pathogenic genes, differential genes were obtained, and on this basis, PPI protein interaction network was constructed, and genes closely related to PFHF in the treatment of inflammation were obtained. In this study, it was found that the key targets of PFHF included IL-6, TNF, IL-1β, INS, CASP3, etc. In addition, CCK-8, ELISA and immunofluorescence experiments proved that PFHF could reduce the levels of IL-6, TNF-α, IL-1β and the protein expression of CASP3, iNOS and Cox-2, and increase the protein expression of INS, thereby alleviating the LPS-induced inflammatory response of RAW264.7 cells.

Under the induction of LPS, RAW264.7 cells release IL-17, and IL-17C then directly acts on the IL-17RE and IL-17RA receptor complex on the cell membrane, activating it to initiate downstream signaling and promote the activation of ACT1 and TRAF6. The activated molecules then further act on NF-κB and AP-1 transcription factors, causing them to enter the nucleus. In the nucleus, these transcription factors promote the transcription of inflammation-related genes such as IL-1β, IL-6, TNF-α and Cox-2 (17). Therefore, the expression level of these genes is increased through the IL-17 signaling pathway, so inhibiting the IL-17 signaling pathway can reduce the expression of inflammatory genes and alleviate related inflammatory responses. Lauffer et al (18)found that an increase in IL-17C levels can amplify the inflammatory response, and depletion of IL-17C can significantly reduce the number of T cells, neutrophils and eosinophils in mouse models of psoriasis and atopic eczema, and lead to significant downmodulation of inflammatory mediators in human skin biopsies of psoriasis and atopic eczema *in vitro*.

The IL-1β gene expression level is elevated, and the IL-1 released by it binds tightly to the IL-1R receptor, triggering the interaction between the IL-1R receptor and IRAK1/4. Subsequently, IRAK1/4 binds to IRAF2/6 and MyD88, and this series of intermolecular interactions activates the ubiquitin ligase Pellion (19). Next, Ubc13 and UEV1A link to TRAF6, prompting TRAF6 to ubiquitinate. After ubiquitination, TRAF6 further attracts TAB1/2 to bind to TAK1, thereby activating TAK1 (20). Activated TAK1 induces the binding of IKβ to IKγ/NEMO. However, when TNF with increased expression is specifically bound to TNF receptor, the TNF receptor closely binds to TRADD and TRAF2/5. In addition, insulin, as a growth factor, binds to the growth factor receptor on the cell membrane to promote the expression and activation of Ras gene. At this time, the expression level of PI3K was affected by TNFR closely bound to TRADD and TRAF2/5 and GF-Rs after promoting RAS activation. The enhanced expression of PI3K then activates the downstream PDK1, resulting in a significant increase in Akt kinase levels (21). The increased expression of Akt produces a series of chain reactions within the cell. Akt indirectly acts on IKα, promoting its binding to IKγ/NEMO. Finally, the IKKβ, IKKα, IKKγ/NEMO complex then activates IκBα for ubiquitination and further binds it to NF-κB2 p52 and p65/RelA. Under the action of kinases such as PKA C and CK2, Indirectly promote the entry of the NF-κB2 p52 and p65/RelA complexes into the nucleus. In the nucleus, p65/RelA, which carries adenylate cyclase, reacts with NF-κB2 p52 to adenylate cyclase, ultimately triggering an inflammatory response. Cheng et al (22) studied the mechanism of glycolysis in microglia activation and neuroinflammation and found that the use of glycolysis inhibitors, Inhibition of phosphorylation of IKβ and IκBα, NF-κB p65 subunit nuclear translocation, and NF-κB transcription activity can significantly inhibit the LPS-induced target of rapamycin mTOR, thereby improving neuroinflammatory diseases associated with microglial activation.

Notarte et al (23)demonstrated that UaB attenuates LPS-induced inflammation by inhibiting NF-κB nuclear translocation and enhancing NRF2 activity, thereby reducing the expression of proinflammatory cytokines (e.g., TNF-α, IL-6, IL-1β) and mediators (e.g., NO, PGE_2_). In contrast, our findings indicate that PFHF, although similarly enriched in flavonols such as rutin, exerts its anti-inflammatory effects primarily through the IL-17 and TNF signaling pathways, rather than directly modulating the NF-κB/NRF2 axis. This suggests that flavonol-enriched fractions from different plant sources may engage distinct inflammatory pathways. The specific regulation of the IL-17 pathway by PFHF provides a novel perspective on its anti-inflammatory mechanism and complements existing research on flavonoid-mediated anti-inflammation.

The results of this study showed that the expression levels of IL-1β and TNF-α in RAW264.7 inflammatory cells induced by LPS were increased, and the levels of IL-1β and TNF-α were decreased after the intervention of PFHF, suggesting that PFHF may inhibit the expression of IL-1β and TNF-α through the inhibition of IL-17 signaling pathway, and play an anti-inflammatory role. However, insulin can control the expression of INS by controlling the upstream components of INS, such as PDX1, MafA and NeuroD1 (24). When insulin secretion increases, negative feedback inhibits upstream elements of INS transcription, resulting in decreased INS expression (25). Coppo et al (26)found that in obese subjects suffering from low-grade inflammation and insulin resistance, cytokine serum level and T cell RAS gene expression were inversely proportional to insulin serum concentration, indicating that the less insulin in serum, the more insulin bound to GF-Rs to deactivate RAS, and the more inflammatory cytokines in serum. Which triggers an inflammatory response. Therefore, it can be inferred that the decrease of INS expression indirectly leads to inflammatory response. This study also found that the expression level of INS protein in RAW264.7 inflammatory cells induced by LPS significantly decreased through immunofluorescence experiment, and the level of INS protein increased after PFHF intervention.

When IL-6 levels rise, it binds to the specific receptor gp130 on the cell membrane, interacts and triggers the activation of intracellular signaling pathways (27). This binding results in phosphorylation of signal transduction and Stat3. Phosphorylated Stat3 is then transported to the nucleus, where it functions as a transcription factor and acts on DNA binding sites such as ISRE/GAS, thereby regulating transcription expression of downstream genes and inducing inflammation (28). The results of this study showed that the expression level of IL-6 in RAW264.7 inflammatory cells induced by LPS was increased, and the level of IL-6 was decreased after PFHF intervention, suggesting that PFHF may inhibit the expression of IL-6 through the inhibition of IL-17 signaling pathway, and play an anti-inflammatory role.

At the same time, when the cells are stimulated by the outside world, the release of IL-17E binds specifically to the IL-17RA and IL-17RB receptors on the target cell membrane, triggering the activation of TRADD. Subsequently, TRADD further activates FADD, a series of signal transduction that ultimately promotes the expression of Casp (29), thereby triggering the process of apoptosis. When Casp is abnormally expressed, cells are abnormally activated, and then deviate from the normal apoptotic path and die in the form of necrosis (30). This non-apoptotic mode of cell death releases a range of intracellular substances that trigger and exacerbate inflammatory responses, adversely affecting tissue homeostasis (31). The immunofluorescence results of this study also showed that the expression level of CASP3 protein in RAW264.7 inflammatory cells induced by LPS increased, and its expression decreased after PFHF intervention, suggesting that PFHF may achieve anti-inflammatory effects by inhibiting CASP3 expression.

Cox-2 is an effector protein downstream of IL-17 signaling pathway, and its protein expression increases after activation through IL-17 signaling pathway. With the increased expression of Cox-2, it catalyzes the conversion of arachidonic acid into prostaglandins, especially PGE2 (32). PGE2 is an important inflammatory mediator, which can increase the permeability of blood vessels and lead to local congestion and edema, thus promoting inflammatory response (33). At the same time, it can also act on nerve endings, causing pain and fever reactions (34). It causes some autoimmune diseases such as rheumatoid arthritis (35). In this study, immunofluorescence experiments showed that the expression level of Cox-2 protein in RAW264.7 inflammatory cells induced by LPS was increased, and its expression was decreased after PFHF intervention, indicating that PFHF may play an anti-inflammatory and analgesic role by inhibiting the expression of Cox-2, an effector protein downstream of IL-17 signaling pathway.

In addition, in the IL-17 signaling pathway, IL-17A and IL-17F bind to the IL-17RA and IL-17RC receptor complexes on the cell surface. After receptor binding, stimulus signals are transduced through a series of adaptor proteins NF-κB and signaling molecules TRAF6 and IKK complexes, thereby activating downstream signaling pathways NF-κB and MAPK pathways (36). Activation of the downstream signaling pathway leads to the activation or nuclear translocation of specific transcription factors NF-κB, AP-1 and C/EBP-β, which binds to the promoter region of iNOS gene, thereby initiating the transcription of iNOS gene (37). The mRNA generated by transcription is then translated into iNOS protein. Once expressed, iNOS protein catalyzes the conversion of arginine into NO and citrulline in the cell, thus playing its role in inflammatory response. Therefore, inhibiting the expression of iNOS can play an anti-inflammatory effect (38). However, this study just found that the expression of iNOS protein in LPS-induced inflammatory cells of RAW264.7 decreased after PFHF intervention, so it can be inferred that one of the anti-inflammatory mechanisms of PFHF is to inhibit the expression of iNOS, an effector molecule downstream of IL-17 signaling pathway.

In this study, it was predicted through network pharmacology that rutin and other components in PFHF could exert anti-inflammatory effects on gene targets such as IL-6, TNF, IL-1β, INS, and CASP3 through IL-17, TNF, and NOD-like receptor signaling pathways. Molecular docking results showed that the key active ingredients in PFHF and the key targets in the PPI network can produce relatively strong protein-binding activity, reflecting the multi-target characteristic of Traditional Chinese Medicine treatment.

## 5 Study limitations

There are several limitations in this study that should be acknowledged. Firstly, in the network pharmacology section of this study, the number of components of PFHF (Total Flavonoids from Polygala fallax Hemsl.) obtained through literature research was limited, and subsequent experiments also failed to fully reflect the anti-inflammatory effects of PFHF. Therefore, there is a need to further explore the active components of PFHF using high-performance liquid chromatography-tandem mass spectrometry. Secondly, the anti-inflammatory effects of PFHF and its potential mechanisms predicted via network pharmacology have only been validated in cell models (*in vitro*). Although these findings are encouraging, the lack of *in vivo* evidence restricts the direct translation of these results to physiological contexts. Future studies should employ established *in vivo* inflammation models, such as the LPS-induced mouse septic shock or carrageenan-induced paw edema model, to confirm the efficacy and safety of PFHF. These experiments are crucial for evaluating systemic effects, bioavailability, and potential toxicity, ultimately providing a more solid foundation for clinical applications. While rutin demonstrated strong binding affinities to several core targets (IL-6, IL-1β, INS, CASP3) and was quantified as a constituent, the contribution of other identified flavonoids (e.g., kaempferol and quercetin derivatives like kaempferol 3-O-beta-D-glucopyranoside, quercetin-3-O-β-D-glucoside) to the overall anti-inflammatory effect cannot be ruled out. These compounds are well-documented in literature for their anti-inflammatory properties and may act synergistically with rutin, targeting different nodes within the inflammatory network (e.g., IL-17, TNF pathways). Our study focused on validating the network predictions and the role of the total extract; future work should involve comparative studies of individual compounds and their combinations to determine if rutin is the primary active component or if the efficacy arises from a synergistic interplay among multiple flavonoids in PFHF.

## Data Availability

The original contributions presented in the study are included in the article/supplementary material. Further inquiries can be directed to the corresponding authors.

## References

[1] Wang ZJ Zhang W Pan X. Determination of rutin in the leaves of dragon blood of Hainan, a Dai medicinal material by HPLC. J Med Pharm Chin Minor. (2018) 24:30–2.

[2] Lin G Pan ZH Ning DS Lin LC Fu YX Li HY. Isolation, identification and antioxidant activity of Polygala fallax Hemsl from the flower of Water Lily. Guihaia. (2022) 42:790–5.

[3] Ru J Li P Wang J Zhou W Li B Huang C. TCMSP: a database of systems pharmacology for drug discovery from herbal medicines. J Cheminform. (2014) 6:13. doi: 10.1186/1758-2946-6-13, PMID: 24735618 PMC4001360

[4] The UniProt Consortium. UniProt: the universal protein knowledgebase in 2023. Nucleic Acids Res. (2023) 51:D523–31. doi: 10.1093/nar/gkac1052, PMID: 36408920 PMC9825514

[5] Sayers EW Beck J Bolton EE Bourexis D Brister JR Canese K. Database resources of the national center for biotechnology information. Nucleic Acids Res. (2024) 52:D43. doi: 10.1093/nar/gkad1044, PMID: 37994677 PMC10767890

[6] Bragina ME Daina A Perez MAS Michielin O Zoete V. The swiss similarity 2021 webTool: novel chemical libraries and additional methods for an enhanced ligand-based virtual screening experience. Int J Mol Sci. (2022) 23. doi: 10.3390/ijms23020811, PMID: 35054998 PMC8776004

[7] Safran M Rosen N Twik M BarShir R Stein TI Dahary D. The GeneCards Suite: Practical Guide to Life Science Databases. In: Practical Guide to Life Science Databases Singapore: Springer Nature (2022). p. 27–56.

[8] Amberger JS Bocchini CA Schiettecatte F Scott AF Hamosh A. OMIM.org: Online Mendelian Inheritance in Man (OMIM®), an online catalog of human genes and genetic disorders. Nucleic Acids Res. (2015) 43:D789–98. doi: 10.1093/nar/gku1205, PMID: 25428349 PMC4383985

[9] Piñero J Saüch J Sanz F Furlong LI. The DisGeNET cytoscape app: Exploring and visualizing disease genomics data. Comput Struct Biotechnol J. (2021) 19:2960–7. doi: 10.1016/j.csbj.2021.05.015, PMID: 34136095 PMC8163863

[10] Knox C Wilson M Klinger CM Franklin M Oler E Wilson A. DrugBank 6.0: the drugBank knowledgebase for 2024. Nucleic Acids Res. (2024) 52:D1265–75. doi: 10.1093/nar/gkad976, PMID: 37953279 PMC10767804

[11] Morris GM Huey R Lindstrom W Sanner MF Belew RK Goodsell DS. AutoDock 4 and AutoDockTools 4: automated docking with selective receptor flexibility. J Comput Chem. (2009) 30:2785–91. doi: 10.1002/jcc.21256, PMID: 19399780 PMC2760638

[12] Abraham J Murtola T Schulz R Páll S Smith JC Hess B. GROMACS: High performance molecular simulations through multi-level parallelism from laptops to supercomputers. SoftwareX. (2015) 1-2:19–25. doi: 10.1016/j.softx.2015.06.001

[13] Maier JA Martinez C Kasavajhala K Wickstrom L Hauser KE Simmerling C. ff14SB: improving the accuracy of protein side chain and backbone parameters from ff99SB. J Chem Theory Comput. (2015) 11:3696–713. doi: 10.1021/acs.jctc.5b00255, PMID: 26574453 PMC4821407

[14] Hess B Bekker H Berendsen HJC Fraaije JGEM. LINCS: A linear constraint solver for molecular simulations. J Comput Chem. (1997) 18:1463–72. doi: 10.1002/(SICI)1096-987X(199709)18:12<1463::AID-JCC4>3.0.CO;2-H

[15] Kang X Chen Y Xin X Liu M Ma Y Ren Y. Human amniotic epithelial cells and their derived exosomes protect against cisplatin-induced acute kidney injury without compromising its antitumor activity in mice. Front Cell Dev Biol. (2021) 9:752053. doi: 10.3389/fcell.2021.752053, PMID: 35186944 PMC8851426

[16] Zhou Y Huang B Zhang Q Yu Y Xiao J. Modeling of new markers for the diagnosis and prognosis of pancreatic cancer based on the transition from inflammation to cancer. Transl Cancer Res. (2024) 13:1425–42. doi: 10.21037/tcr-23-1365, PMID: 38617519 PMC11009810

[17] Facchin BM Dos Reis GO Vieira GN Mohr ETB da Rosa JS Kretzer IF. Inflammatory biomarkers on an LPS-induced RAW 264.7 cell model: a systematic review and meta-analysis. Inflammation Res. (2022) 71:741–58. doi: 10.1007/s00011-022-01584-0, PMID: 35612604

[18] Lauffer F Jargosch M Baghin V Krause L Kempf W Absmaier-Kijak M. IL-17C amplifies epithelial inflammation in human psoriasis and atopic eczema. J Eur Acad Dermatol Venereol. (2020) 34:800–9. doi: 10.1111/jdv.16126, PMID: 31793105

[19] Murphy M Xiong Y Pattabiraman G Qiu F Medvedev AE. Pellino-1 positively regulates toll-like receptor (TLR) 2 and TLR4 signaling and is suppressed upon induction of endotoxin tolerance. J Biol Chem. (2015) 290:19218–32. doi: 10.1074/jbc.M115.640128, PMID: 26082489 PMC4521043

[20] Sanjo H Takeda K Tsujimura T Ninomiya-Tsuji J Matsumoto K Akira S. TAB2 is essential for prevention of apoptosis in fetal liver but not for interleukin-1 signaling. Mol Cell Biol. (2003) 23:1231–8. doi: 10.1128/MCB.23.4.1231-1238.2003, PMID: 12556483 PMC141141

[21] Bamodu OA Chang HL Ong JR Lee WH Yeh CT Tsai JT. Elevated PDK1 expression drives PI3K/AKT/MTOR signaling promotes radiation-resistant and dedifferentiated phenotype of hepatocellular carcinoma. Cells. (2020) 9:746. doi: 10.3390/cells9030746, PMID: 32197467 PMC7140693

[22] Cheng J Zhang R Xu Z Ke Y Sun R Yang H. Early glycolytic reprogramming controls microglial inflammatory activation. J Neuroinflammation. (2021) 18:129. doi: 10.1186/s12974-021-02187-y, PMID: 34107997 PMC8191212

[23] Notarte KIR Quimque MTJ Macaranas IT Bajardo KJL Dacanay AT Burlaza MJB. Attenuation of Lipopolysaccharide-Induced Inflammatory Responses through Inhibition of the NF-κB Pathway and the Increased NRF2 Level by a Flavonol-Enriched n-Butanol Fraction from Uvaria alba. ACS Omega. (2023) 8:5377–92. doi: 10.1021/acsomega.2c06451, PMID: 36816691 PMC9933231

[24] Docherty HM Hay CW Ferguson LA Barrow J Durward E Docherty K. Relative contribution of PDX-1, MafA and E47/beta2 to the regulation of the human insulin promoter. Biochem J. (2005) 389:813–20. doi: 10.1042/BJ20041891, PMID: 15862113 PMC1180732

[25] Jing G Chen J Xu G Shalev A. Islet ChREBP-β is increased in diabetes and controls ChREBP-α and glucose-induced gene expression via a negative feedback loop. Mol Metab. (2016) 5:1208–15. doi: 10.1016/j.molmet.2016.09.010, PMID: 27900263 PMC5123192

[26] Coppo M Bandinelli M Chiostri M Modesti PA Poggesi L Boddi M. T cell-based RAS activity and insulin levels in obese subjects with low grade inflammation. Am J Med Sci. (2021) 363:428–34. doi: 10.1016/j.amjms.2021.09.003, PMID: 34571038

[27] Rose-John S Jenkins BJ Garbers C Moll JM Scheller J. Targeting IL-6 trans-signalling: past, present and future prospects. Nat Rev Immunol. (2023) 23:666–81. doi: 10.1038/s41577-023-00856-y, PMID: 37069261 PMC10108826

[28] Adach A Ellert-Miklaszewska A Kaminska B. Molecular characterization of STAT signaling in inflammation and tumorigenesis. Methods Mol Biol. (2009) 512:265–78. doi: 10.1007/978-1-60327-530-9_14, PMID: 19347282

[29] Yang Z Wang S Liu H Xu S. MAPK/iNOS pathway is involved in swine kidney necrosis caused by cadmium exposure. Environ pollut. (2021) 274:116497. doi: 10.1016/j.envpol.2021.116497, PMID: 33540250

[30] Love S Barber R Wilcock GK. Neuronal death in brain infarcts in man. Neuropathol Appl Neurobiol. (2000) 26:55–66. doi: 10.1046/j.1365-2990.2000.00218.x, PMID: 10736067

[31] Weinlich R Oberst A Beere HM Green DR. Necroptosis in development, inflammation and disease. Nat Rev Mol Cell Biol. (2017) 18:127–36. doi: 10.1038/nrm.2016.149, PMID: 27999438

[32] Bai M Zhang L Fu B Bai J Zhang Y Cai G. IL-17A improves the efficacy of mesenchymal stem cells in ischemic-reperfusion renal injury by increasing Treg percentages by the COX-2/PGE2 pathway. Kidney Int. (2018) 93:814–25. doi: 10.1016/j.kint.2017.08.030, PMID: 29132705

[33] Das UN. Essential fatty acids and their metabolites in the pathobiology of inflammation and its resolution. Biomolecules. (2021) 11:1873. doi: 10.3390/biom11121873, PMID: 34944517 PMC8699107

[34] Ma W St-Jacques B Duarte PC. Targeting pain mediators induced by injured nerve-derived COX2 and PGE2 to treat neuropathic pain. Expert Opin Ther Targets. (2012) 16:527–40. doi: 10.1517/14728222.2012.680955, PMID: 22519838

[35] Akaogi J Nozaki T Satoh M Yamada H. Role of PGE2 and EP receptors in the pathogenesis of rheumatoid arthritis and as a novel therapeutic strategy. Endocr Metab Immune Disord Drug Targets. (2006) 6:383–94. doi: 10.2174/187153006779025711, PMID: 17214584

[36] Yilmaz DE Senol SP Temiz-Resitoglu M Sahan-Firat S Tunctan B. NLRX1 ligand, docosahexaenoic acid, ameliorates LPS-induced inflammatory hyperalgesia by decreasing TRAF6/ IKK/ IkB-a/ NF-kB signaling pathway activity. Cell Mol Biol (Noisy-le-grand). (2023) 69:15–23. doi: 10.14715/cmb/2023.69.9.3, PMID: 37807339

[37] Roodgar M Ross CT Kenyon NJ Marcelino G Smith DG. Inducible nitric oxide synthase (iNOS) regulatory region variation in non-human primates. Infect Genet Evol. (2015) 31:236–44. doi: 10.1016/j.meegid.2015.01.015, PMID: 25675838 PMC4361290

[38] Grottelli S Amoroso R Macchioni L D'Onofrio F Fettucciari K Bellezza I. Acetamidine-Based iNOS Inhibitors as Molecular Tools to Counteract Inflammation in BV2 Microglial Cells. Molecules. (2020) 25:2646. doi: 10.3390/molecules25112646, PMID: 32517272 PMC7321217

